# Nocturnal periodicity of *Phlebotomus* (*Larroussius*) *orientalis* (Diptera: Psychodidae) in an endemic focus of visceral leishmaniasis in Northern Ethiopia

**DOI:** 10.1186/s13071-015-0804-7

**Published:** 2015-03-28

**Authors:** Araya Gebresilassie, Oscar David Kirstein, Solomon Yared, Essayas Aklilu, Aviad Moncaz, Habte Tekie, Meshesha Balkew, Alon Warburg, Asrat Hailu, Teshome Gebre-Michael

**Affiliations:** Department of Zoological Sciences, Addis Ababa University, Addis Ababa, Ethiopia; Department of Biology, College of Natural Science, Jigjiga University, Jigjiga, Ethiopia; Department of Microbiology and Molecular Genetics, The Institute of Medical Research Israel-Canada The Kuvin Center for the Study of Infectious and Tropical Diseases, Faculty of Medicine, The Hebrew University, Hadassah Medical School, Jerusalem, Israel; Aklilu Lemma Institute of Pathobiology, Addis Ababa University, Addis Ababa, Ethiopia; Department of Microbiology, Immunology and Parasitology, College of Health Sciences, Addis Ababa University, Addis Ababa, Ethiopia

**Keywords:** Nocturnal periodicity, *Phlebotomus orientalis*, Tahtay Adiyabo, Visceral leishmaniasis

## Abstract

**Background:**

*Phlebotomus orientalis* is the major vector of the intramacrophage protozoa, *Leishmania donovani*, the etiological agent of visceral leishmaniasis (VL) in northern Ethiopia and Sudan. The objective of this study was to determine the nocturnal periodicity of *P. orientalis* in the VL endemic focus of Tahtay Adiyabo district, northern Ethiopia.

**Methods:**

Sandflies were collected using CDC light traps by changing collecting bags at an hourly interval from dusk to dawn for six months (January-June 2013) from outdoors (i.e. peri-domestic and agricultural fields). Sandfly specimens collected in the study were identified to species level and counted.

**Results:**

In total, 21,716 nocturnally active sandfly specimens, which belong to two genera (i.e., *Phlebotomus* and *Sergentomyia*) were collected and identified. In the collection, *P. orientalis*, the dominant species in the genus *Phlebotomus*, constituted 33.79% while *Sergentomyia* spp. comprised 65.44%. Analysis of data showed that activity of *P. orientalis* females increased from 18:00 to 24:00 hours, with a peak after midnight (24:00–03:00 hrs). Likewise, activity of parous *P. orientalis* females was found to be unimodal, peaking at 24–01:00 hrs.

**Conclusion:**

*P. orientalis* females had marked nocturnal activity, which peak after midnight. Similarly, the epidemiologically dangerous parous females generally were more active after midnight. Therefore, humans are at risk of *L. donovani* infections through the bite of *P. orientalis* possibly between midnight and dawn.

## Background

Phlebotomine sandflies have considerable public health importance in the tropics and subtropics attributed mainly to their role as potent vectors of the various forms of leishmaniasis (visceral and dermal), bartonellosis and 3-day fever (Pappataci fever). These diseases are transmitted by the bite of infected female sandflies of the genus *Phlebotomus* in the Old World and *Lutzomyia* in the New World when taking repetitive bloodmeals [1-4].

Most phlebotomine sandflies from the Old World are active during the night [2,5-7]. It is this time that adult sandflies exhibit predominant activity periods for behaviors such as sugar feeding, host seeking, blood feeding, mating, and oviposition [8-11]. However, species-specific differences are observed in the peak activity, which can influence the vectorial capacity of different species [5,12,13]. Little is known, nonetheless, why the time of nocturnal activity of most sandflies varies with season and time.

Information on the nocturnal activity and biting rhythms of *P. orientalis* is available from Sudan and two works from north-west Ethiopia [14-17]. In Sudan, Quate [14] indicated that peak biting densities of *P. orientalis* took place between 18:30 and 20:30 hours. In eastern Sudan, the hourly light trap and human-landing collections of *P. orientalis* continued until late in the night [18]. Earlier, Gebre-Michael and Lane [19] studied the nocturnal periodicity of *P. martini* and *P. celiae* in southern Ethiopia. Nevertheless, this has not been systematically investigated on populations of *P. orientalis* in northern Ethiopia.

Knowledge on the sandfly nocturnal activity is noteworthy because it indicates the time when a person is most likely to be bitten by the sandfly vector and possibly get leishmaniasis. It also reveals the best possible time to collect and monitor the adults. As well, information on the peak activity period of sandfly vectors can be used to schedule outdoor activities to avoid peak exposure periods. In this perspective, the current study was designed to observe the nocturnal activity patterns of *P. orientalis* in an endemic focus of VL in Tahtay Adiyabo district, northern Ethiopia. Effects of variations in hourly nighttime temperature and relative humidity on the nocturnal activity patterns of the vector species were also studied.

## Methods

### Study area

Entomological investigation was conducted in the locality of Geza Adura within the village of Lemlem in the Tahtay Adiyabo district (14°22′27″N/ 37°44′36″E) in the Tigray Regional State, Northern Ethiopia. The administrative center of the district is located 1,117 km north of Addis Ababa and 402 km north-west of Mekelle, the capital of Tigray Regional State. The area is lowland plains with an average altitude of 1,028 meters above sea level. The climate is generally sub-tropical-arid, with an extended dry period of nine to ten months. The area has a uni-modal pattern of rainfall (July-September) with a mean annual precipitation of about 600 mm. March to May is the hottest part of the year with an average temperature of 39°C at noon and January is the coldest one with an average temperature of 14.2°C at night.

The village is situated on rocky hill surrounded by large farm fields of vertisols alternating with large tracts of red clay soil. The inhabitants are mainly engaged in the production of cereals and oilseeds and raising domestic animals.

### Sandfly collection

Sandflies were sampled using two CDC light traps from outdoors (viz., peri-domestic and agricultural fields) bimonthly for 6 months (between January 2013 and June 2013), when the sandfly abundance was high. Traps were set and their collecting bags were replaced at hourly intervals starting before sunset till after sunrise (18:00–07:00 hrs). The removed collection bags were replaced by another set of bags for the next one hour. The next morning, captured sandflies were transported to the laboratory where *P. orientalis* females were sorted out from males and the rest of *Phlebotomus* and *Sergentomyia* spp. Male *Phlebotomus* and *Sergentomyia* spp. were preserved in 70% ethanol for later species identification.

### Determination of abdominal status and parous rates of female *P. orientalis*

The trapped *P. orientalis* females were examined for abdominal status and the numbers of unfed, freshly blood-fed, half-gravid, and gravid sandflies were recorded. After that, representative samples of unfed females of *P. orientalis* were dissected under a dissecting microscope to determine parous rates (reproductive history) and gonotrophic states. For this purpose, a few drops of normal saline were added on a glass slide, the ovaries were dissected out using a pair of fine dissecting needles. The ovaries were then examined under a phase contrast microscope at 40 × 10 magnifications. The accessory glands contained some granules if the sandfly was parous, but contained no granules if nulliparous [20].

### Sandfly identification

Collected sandflies were mounted on microscope slides in Hoyer’s medium with their heads separate from thoraces and abdomens. Species were identified based on the morphology of the external genitalia of males and the pharynx, antennal features and spermathecae of females, using different keys, [14,21] and other publication [22].

### Meteorological data recording

Variations in hourly temperature and relative humidity (RH) were recorded using data loggers (HOBO Micro Station©) on hourly basis during the collection nights.

### Data analysis

Prior to data analysis, sandfly numbers were checked for normality by 1-Sample Kolmogorov - Smirnov Z test (K-S). One-way ANOVA was used to compare the overall (male and female) hourly activity patterns of *P. orientalis* during the night. Tukey’s Studentized test *post hoc* analysis was utilized for mean separation where ANOVA was significant. The non-parametric equivalent test (Kruskal-Wallis) was used to compare the hourly activity of individual male and female *P. orientalis* populations. Similarly, to assess differences in parous and blood feeding rates among collection intervals, a Kruskal-Wallis test was followed. For non-parametric comparisons, multiple-Mann–Whitney *U*-test was used and, *p*-values were adjusted with the Bonferroni correction to adjust for the inflation of type I errors when several Mann–Whitney tests are performed [23]. Spearman’s rank-correlation analysis (*P* < 0.05) was also used to compare the effects of average nighttime temperature and humidity on the number of flies captured per hour. Statistical analysis were considered significant when *P* < 0.05 unless stated. All statistical analyses were carried out using IBM SPSS statistics, version 19 for Windows (SPSS Inc., Chicago, IL, USA) and Microsoft® Office Excel 2007.

## Results

### Nocturnal activity rhythms

A total of 21,716 (65.20% male) nocturnally active sandfly specimens, which belong to two genera were collected and identified. In the collection, *P. orientalis*, the dominant species in the genus *Phlebotomus*, constituted 33.79% while *Sergentomyia* spp. comprised 65.44% (Table 1).

**Table 1:** **Nocturnally active sandfly species captured using CDC light traps in Tahtay Adiyabo district, January-June 2013**

	**Number of sandflies collected**			
**Sandfly Species**	**Male**	**Female**	**Total**	**Relative frequency (%)**
*P. orientalis*	5,343	1,995	7,338	33.79
*P. bergeroti*	54	17	71	0.33
*P. lesleyae*	9	20	29	0.13
*P. rodhaini*	6	15	21	0.09
*P. heischi*	6	11	17	0.08
*P. duboscqi*	7	8	15	0.07
*P. martini*	3	6	9	0.04
*P. papatasi*	1	2	3	0.01
*P. alexandri*	0	1	1	0.005
*Sergentomyia* spp.	8,729	5,483	14,212	65.44
Total	14,158	7,558	21,716	100

**Figure 1:**
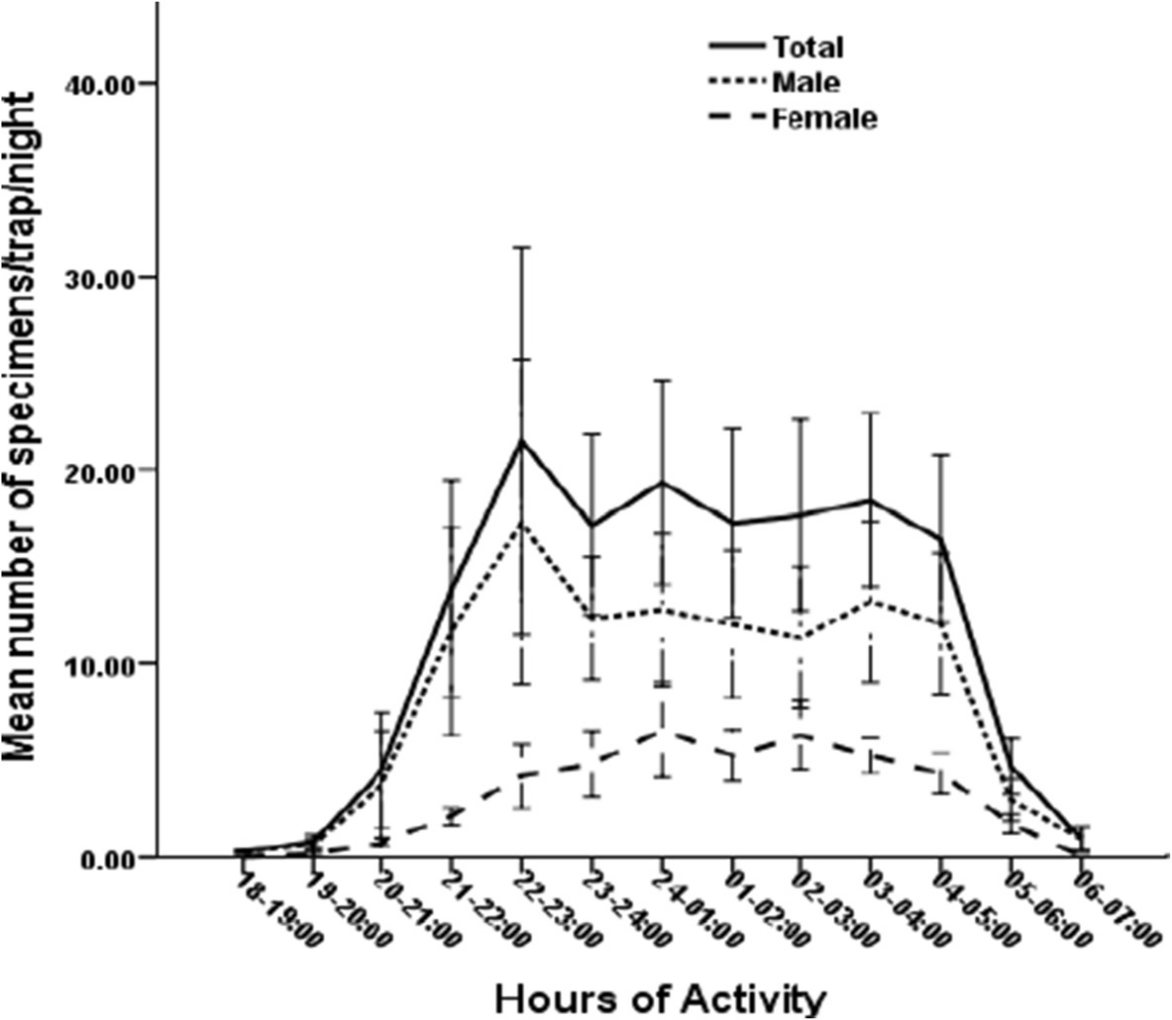
**Nocturnal activity patterns of female and male** ***P. orientalis.***

The overall hourly activity patterns of *P. orientalis* was significantly different among collection intervals (ANOVA, F_(df=12)_ =8.04; *P* = 0.000, Figure 1) with a peak nocturnal activity (21.5 flies/trap/hr) before midnight (22:00–23:00 hrs). High fly activity continued after midnight until a smaller peak towards the morning (04:00–05:00 hrs) after which sharply declined (Figure 1). The Kruskal-Wallis test also indicated a significant difference in the mean number of male (*P =* 0.000) and female (*P =* 0.000) *P. orientalis* caught each hour (Figure 1). *P. orientalis* females were lower in number than the males throughout the night, but appeared to increase slowly as the night progressed with a peak just after midnight (24:00–03:00 hrs), after which decreased progressively (Figure 1). Males had a single peak at 22:00–23:00 hrs, though continued to be moderately active until 05:00 hrs (Figure 1).

**Figure 2:**
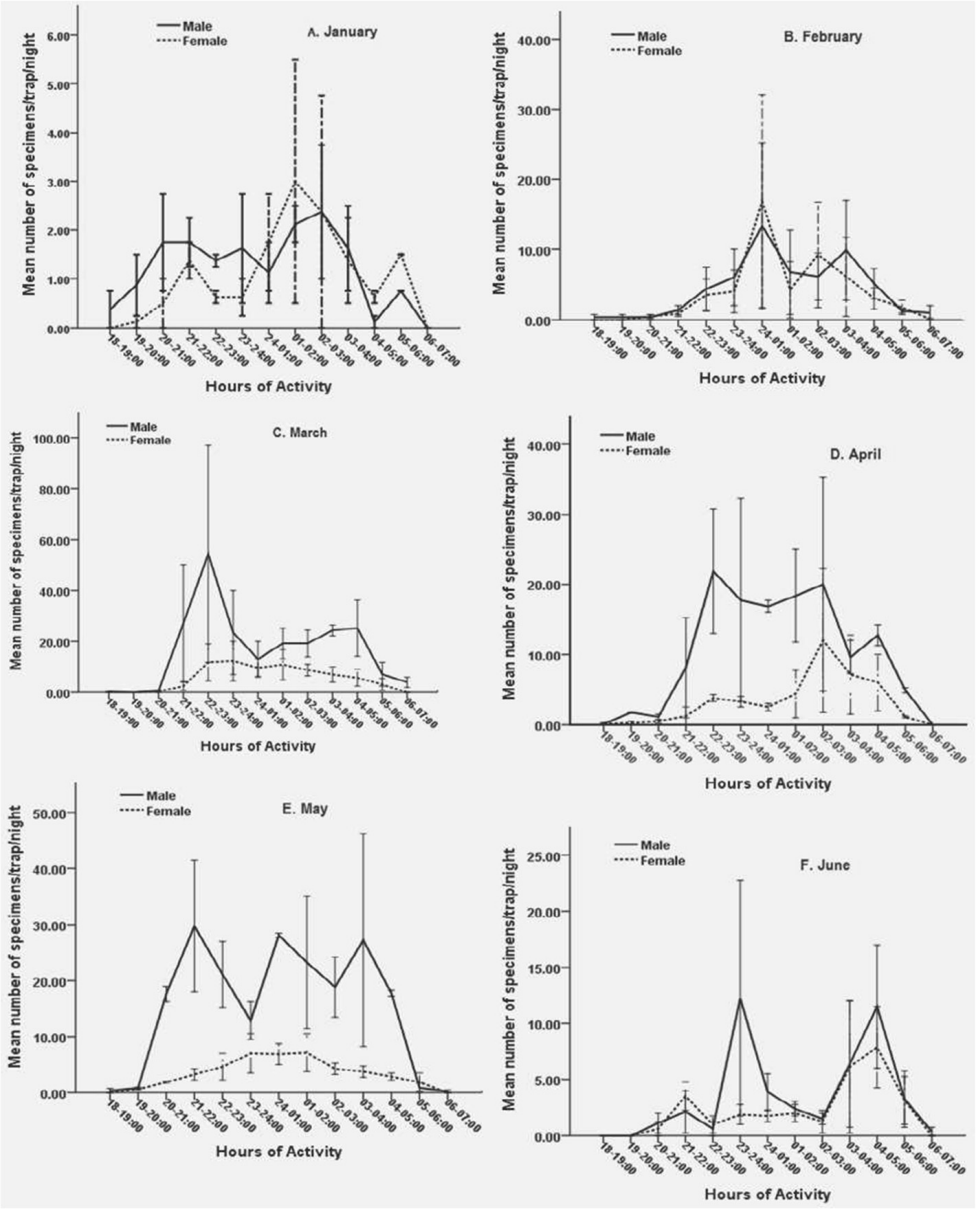
**Hourly activities of male and female** ***P. orientalis*** **in each month. A**: January. **B**: February. **C**: March. **D**: April. **E**: May and **F**: June.

Nonetheless, the exact timing of the peak activity of the females varied with months (Figure 2-A-F). The nocturnal activity rhythm of *P. orientalis* females in January, February, April, and June was similar, showing maximal activity in the second half of the night. On the other hand, the peak nocturnal periodicity of females in March and May was in the first half of the midnight (Figure 2-C and D).

### Abdominal status and parous rates

Abdominal categories for captured *P. orientalis* females are shown in Table 2. Of 1,995 *P. orientalis* females trapped, 1,630 (81.70%) were unfed, 305 (15.29%) were freshly blood-fed, 14 (0.70%) semi-gravid and 46 (2.31%) were gravid.

**Table 2:** **Abdominal status of nocturnally active female** ***P. orientalis*** **determined during January-June 2013**

	**Abdominal status**				
**Hours of Activity**	**Unfed**	**Freshly blood-fed**	**Semi-gravid**	**Gravid**	**Total**
18:00–19:00	1	0	0	0	1
19:00–20:00	7	1	0	0	8
20:00–21:00	29	4	1	1	35
21:00–22:00	61	27	2	12	102
22:00–23:00	157	38	1	6	202
23:00–24:00	177	45	0	5	227
24:00–1:00	265	50	1	4	320
1:00–2:00	205	46	0	2	253
2:00–3:00	264	32	3	4	303
3:00–4:00	220	26	5	1	252
4:00–5:00	172	27	0	10	209
5:00–6:00	71	9	1	1	82
6:00–7:00	1	0	0	0	1
Total	1,630 (81.70%)	305 (15.29%)	14 (0.70%)	46 (2.31%)	1,995

**Figure 3:**
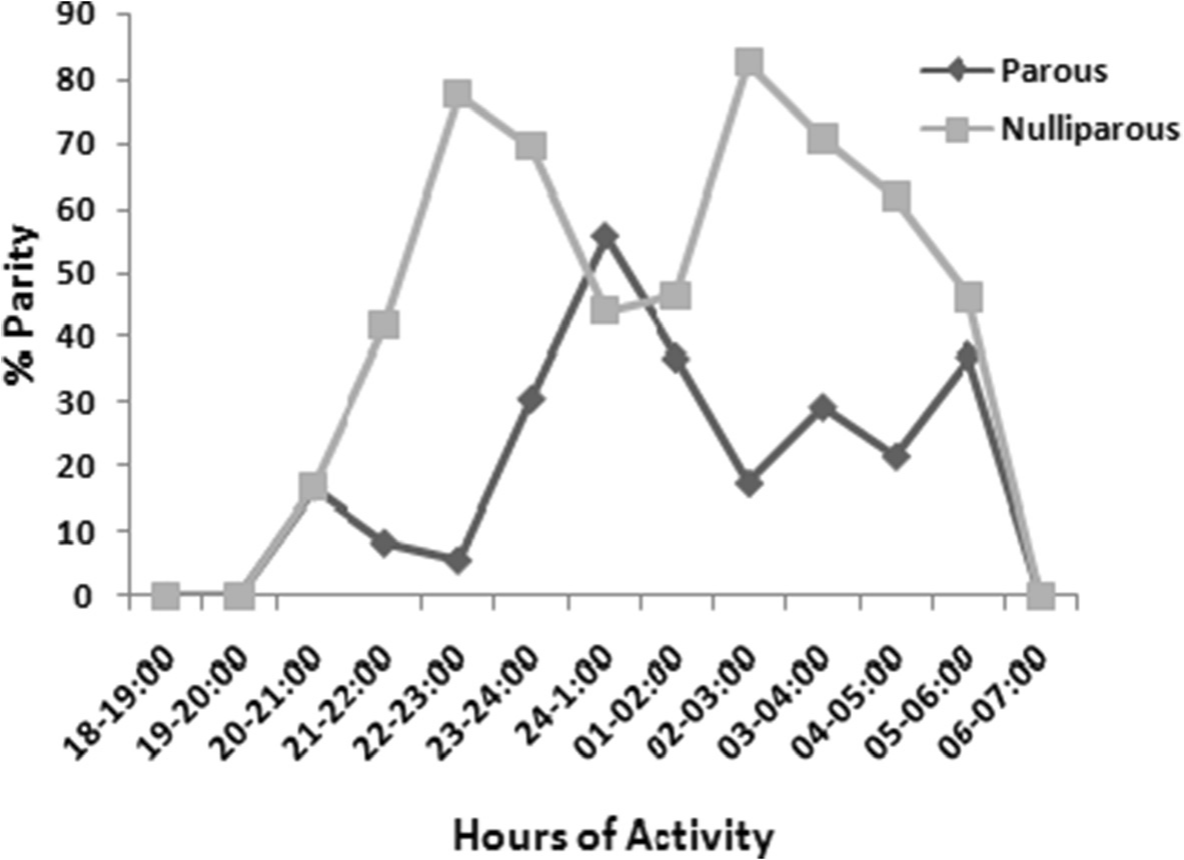
**Hourly proportions of nulliparous and parous** ***P. orientalis*** **females.**

Out of 236 unfed females dissected for parous state, 80 (33.90%) were parous. There was significant difference in the hourly proportion of parous females caught, where the highest (55.90%) being after midnight (24–01:00) (Kruskal-Wallis test, *P* < 0.05, Figure 3). Nulliparous females; however, had bimodal peak activity period: the first between 22:00 and 23:00 hrs and the rest from 02:00–03:00 hrs (Figure 3).

### Blood feeding periods

The number of freshly blood-fed *P. orientalis* females caught during different activity periods is reported in Table 2. Significant differences in the hourly nighttime blood feeding pattern of *P. orientalis* females were observed (Kruskal-Wallis test, *P* = 0.001). Higher proportions (62.29%) of freshly blood-fed females were caught after midnight with peak at 24:00–01:00 hr. However, blood-feeding activity of this species steadily declined after 05:00 hr.

### Effects of temperature and relative humidity on nocturnal activity

**Figure 4:**
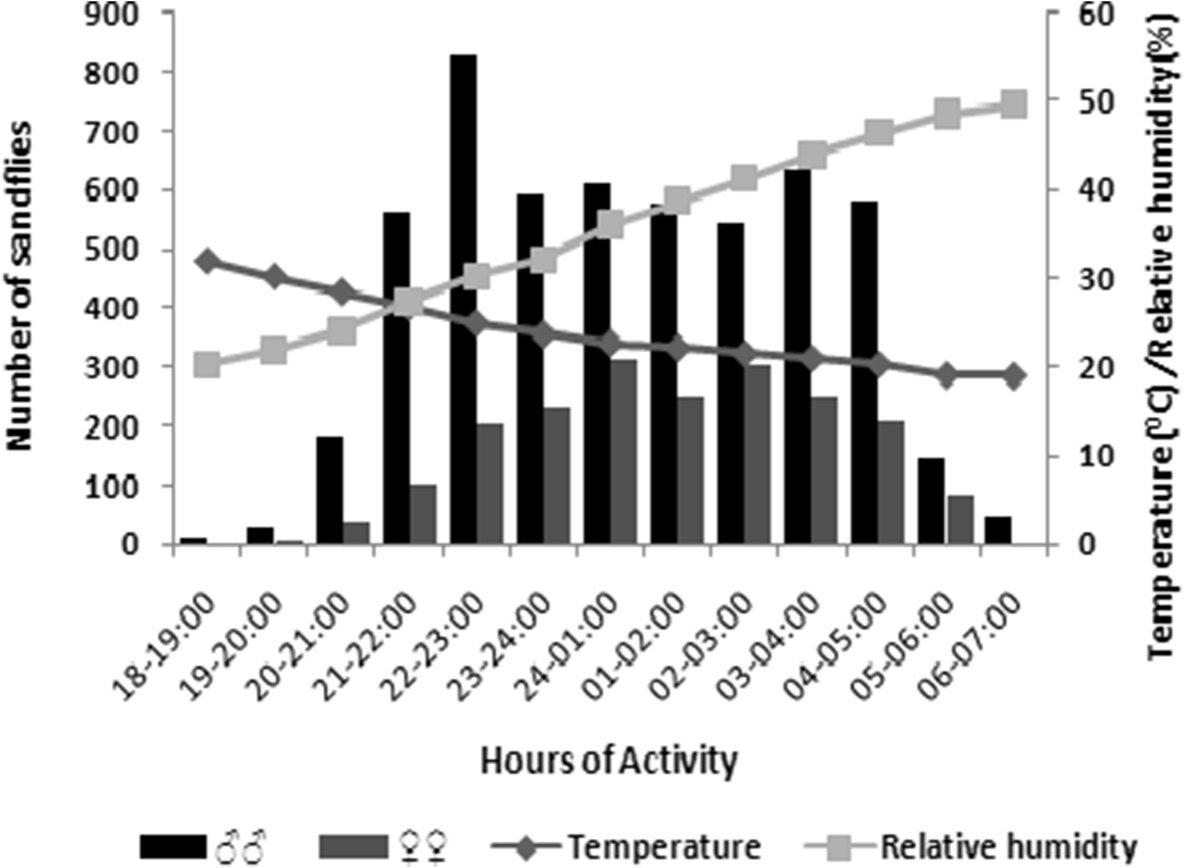
**Nocturnal activity rhythms of male and female** ***P. orientalis*** **relative to average temperature and relative humidity variation during different hours of the night, January-June 2013.**

The relationship between the activity of *P. orientalis* males and females and weather (temperature and relative humidity) is illustrated in Figure 4. Temperature decreased and relative humidity increased after sunset through the night. There is no significant correlation between the number of male *P. orientalis* caught at hourly intervals with the hourly night temperature (r = −0.129; *P* = 0.259) and relative humidity (r = 0.032, *P* = 0.783). Whereas, the nocturnal activity of female *P. orientalis* had a weak negative significant correlation with temperature (r = −0.229, *P* = 0.044) and a weak non-significant positive correlation with relative humidity (r = 0.173, *P* = 0.129).

## Discussion

Different studies have indicated that sandflies are either crepuscular or nocturnal in their diel periodicity, but species-specific differences are observed in the peak activity, which can influence the vectorial capacity of different species [8,13,24].

In the current study, both sexes of *P. orientalis* were found to have nocturnal activity with different patterns. Females appeared to have increased activity during midnight, which corresponded to the relatively low temperature. Thereafter, activity patterns subsided progressively towards dawn before it stopped immediately after sunrise. Our findings are in accordance with the reports of Lemma *et al*. [17] who used CDC light traps and found that the peak the nocturnal activities of female *P. orientalis* were between 24:00 and 1:00 close to animal shelters in Kafta Humera, north-west Ethiopia. However, our results differed from those reported for this species in Sudan based on human landing collections. *P. orientalis* exhibited peak man-biting activity between 20:00 and 22:00 hours in eastern Sudan [16]. Possible explanations for these differences in the pattern of activity peaks are the regional difference in environmental and other endogenous factors related to strain variation, where they were illustrated as factors to govern sandfly nocturnal activities [25]. Secondly, the type of collection methods used could also account for the differences. The results of this study are based on light trap collections, and may not precisely represent the pattern of host seeking *P. orientalis* females if man landing/biting catches were concurrently conducted. Man landing/biting catches could not be conducted because of ethical considerations surrounding the use of human landing catches; however, the data presented in this study could reflect the activity patterns of *P. orientalis*.

The data also showed that females display variations in peak nocturnal activity with different months, which is consistent with observations of Dinesh *et al*. [5] in India and Coleman *et al*. [24] in Southern Iraq. The important factor affecting female nocturnal activity in this study appeared to be the average temperature, though the interaction was not strong. As the temperature decreased from 32 to 24°C, the activity pattern increased rapidly and reached maximum when temperature value was between 23 and 22°C, then, decreased steadily when the temperature was below 19°C. Therefore, detailed studies on the possible effects of cloud cover and wind velocity on the nocturnal activity of sandflies may improve our understanding on those variations.

Males of *P. orientalis* were more active earlier than females and this could be associated with mating behavior. Similar adaptive behaviors were also recognized for males of *Lu. longipalpis* in Colombia [10] and *P. argentipes* in India [5]. The adaptive significance of this early landing of male *P. orientalis* could be for lekking purposes, thereby increasing their chances of mating with female flies that will be attracted to the hosts [10,26,27].

Nulliparous females of *P. orientalis* seemed to have a bimodal peak activity period. The first batch of nulliparous females arrived earlier than parous flies, which corresponded to the period when male abundance was greatest. This implies an overlap in periodicity for mating between the male and newly emerged female populations. Other activities of nulliparous females might be related to attending their physiological demands such as search for sugar and bloodmeal. On the other hand, large proportions of parous females of *P. orientalis* were caught between midnight and dawn. Equally, more numbers of blood-fed individuals were collected after midnight with a peak between 24:00 and 02:00 hours. This might be because females feed soon after oviposition, which was also observed in *Lu. longipalpis* females [28]. However, Ngumbi *et al*. [11] in Kenya noted that more than 58.0% of blood-fed *Sergentomyia schwetzi* was caught before midnight, which was not the case with this study.

Successful bloodmeal acquisition by biting insects requires that their active periods overlap with periods of host availability, predator inactivity, and suitable environmental conditions [29,30]. Therefore, age structure and blood feeding rhythm differences of *P. orientalis* before and after midnight might have epidemiological implications. During the dry season, almost all villagers in the study area sleep outside the house and often go to sleep after 22:00 hr. This sleeping period is not only the time that corresponds to the higher *P. orientalis* female biting rhythms, but also to the time that the human hosts become inactive and less defensive for sandfly blood feeding. At the same time, a consistent use of insecticide treated bed nets among residents who are sleeping outside houses is less (Gebresilassie *et al*., unpublished data), increasing the possibility of people bite to sandfly vectors. The presence of large numbers of parous females after midnight could exacerbate the risk of acquiring VL infection.

## Conclusions

In conclusion, in our focus area, *P. orientalis* females and males showed marked nocturnal periodicity, with a peak between 24:00 and 03:00 hrs and 22:00 and 23:00 hrs, respectively. Female activities were much lower than the males with a peak after midnight. Likewise, the epidemiologically dangerous parous females generally were more active after midnight. While these observations are important as a general precaution against sandfly exposure, the exact activity patterns of host seeking females on human hosts remain to be determined whether it corresponds to the present observation based on light trap catches. The results of this study provide insights to protect better the individual or the community from sandfly bites by the use of repellents or insecticide treated nets (ITNs).
